# Ferroptosis-related long non-coding RNA signature predicts the prognosis of bladder cancer

**DOI:** 10.1186/s12885-022-09805-9

**Published:** 2022-06-30

**Authors:** Jian Hou, Zhenquan Lu, Xiaobao Cheng, Runan Dong, Yi Jiang, Guoqing Wu, Genyi Qu, Yong Xu

**Affiliations:** 1 Department of Urology, Zhuzhou Central Hospital, Zhuzhou, 412007 China; 2 Department of Surgery, Division of Urology, The University of Hongkong-ShenZhen Hospital, Shenzhen, 518000 China

**Keywords:** TCGA, Bladder cancer, Ferroptosis, Long non-coding RNA, Prognosis signature

## Abstract

**Background:**

Ferroptosis is an iron-dependent programmed cell death modality that may have a tumor-suppressive function. Therefore, regulating ferroptosis in tumor cells could serve as a novel therapeutic approach. This article focuses on ferroptosis-associated long non-coding RNAs (lncRNAs) and their potential application as a prognostic predictor for bladder cancer (BCa).

**Methods:**

We retrieved BCa-related transcriptome information and clinical information from the TCGA database and ferroptosis-related gene sets from the FerrDb database. Least absolute shrinkage and selection operator regression (LASSO) and Cox regression models were used to identify and develop predictive models and validate the model accuracy. Finally, we explored the inter-regulatory relationships between ferroptosis-related genes and immune cell infiltration, immune checkpoints, and m^6^A methylation genes.

**Results:**

Kaplan–Meier analyses screened 11 differentially expressed lncRNAs associated with poor BCa prognosis. The signature (AUC = 0.720) could be utilized to predict BCa prognosis. Additionally, GSEA revealed immune and tumor-related pathways in the low-risk group. TCGA showed that the p53 signaling pathway, ferroptosis, Kaposi sarcoma − associated herpesvirus infection, IL − 17 signaling pathway, MicroRNAs in cancer, TNF signaling pathway, PI3K − Akt signaling pathway and HIF − 1 signaling pathway were significantly different from those in the high-risk group. Immune checkpoints, such as PDCD-1 (PD-1), CTLA4, and LAG3, were differentially expressed between the two risk groups. m^6^A methylation-related genes were significantly differentially expressed between the two risk groups.

**Conclusion:**

A new ferroptosis-associated lncRNAs signature developed for predicting the prognosis of BCa patients will improve the treatment and management of BCa patients.

**Supplementary Information:**

The online version contains supplementary material available at 10.1186/s12885-022-09805-9.

## Introduction

Bladder cancer (BCa) is one of the most common malignancies in the urogenital system and one of the top ten predominant malignancies worldwide [1]. Bladder cancer is divided into muscle-invasive bladder cancer (MIBC) and non-muscle-invasive bladder cancer (NMIBC), according to whether the tumor invades the muscle layer of the bladder [2]. Although surgical treatment and postoperative Bacillus Calmette-Guerin (BCG) perfusion and some other immunotherapy are applied to the clinical treatment of BCa [3], about 20% of BCa cases still show an invasion into the bladder muscle. Despite the available treatments, MIBC recurrence, progression, and mortality are high [4]. The five-year overall survival (OS) rate for all stages of urothelial cancer patients is approximately 66–68% [5]. Effective clinical management of BCa is greatly limited by the preclinical models and a lack of accurate biomarkers for early diagnosis. Therefore, it is crucial to explore other forms of cell death to overcome the resistance of tumor cells and discover new and effective prognostic biomarkers for early BCa.

Ferroptosis is a newly discovered form of programmed cell death, operating differently from apoptosis and autophagy [6]. Ferroptosis is iron-dependent because it is triggered by the accumulation of intracellular iron, lipid peroxides, and reactive oxygen species (ROS). The primary mechanism of ferroptosis is the induction of cell death through lipid peroxidation in the presence of divalent iron or ester oxygenase, which catalyzes the high expression of unsaturated fatty acids in cell membrane [7]. Ferroptosis is involved in various critical biological processes, including cancer and neurodegenerative diseases [8, 9]. Furthermore, recent studies have increasingly confirmed that the regulation of ferroptosis could serve as a new therapeutic tool [10], which requires a closer investigation on the link between ferroptosis and cancer.

Long non-coding RNAs (lncRNAs) are a class of non-coding RNA longer than 200 nucleotides in length. LncRNAs can regulate different physiological and biochemical cellular processes via mediating chromosomal modifications, transcriptional activation, and interference [11]. In addition to gene regulation, lncRNAs are involved in various bioregulatory processes, including those related to tumorigenesis, progression, and metastasis [12]. However, current knowledge on the association between ferroptosis, lncRNAs, and cancer is far from comprehensive. Wang et al. showed that in lung cancer, lncRNA LINC00336 regulates tumor progression by inhibiting ferroptosis mechanisms through interaction with ELAVL1 [13]. LncRNA LINK-A, an oncogene, plays a vital role in endogenous tumor suppression and presentation of cancer cell antigens [14]. In addition, the lncRNAMT1DP showed increased sensitivity of non-small cell lung cancer to ferroptosis via regulating the miR-365a-3p/NRF2 signaling pathway [15]. Therefore, lncRNAs can act as an independent prognostic factor for tumors, providing new directions for individualized tumor treatment.

Currently, there are no studies reporting the association of ferroptosis-related lncRNAs with BCa overall survival. This study developed the first prognostic model of differentially expressed ferroptosis-related lncRNAs with prognostic lncRNAs for BCa.

## Method

### Data collection

BCa transcriptome expression data were retrieved from the TCGA portal (https://cancergenome.nih.gov/). The data included 414 tumor samples and 19 healthy samples. Clinical characteristics of the BCa patients obtained included age, stage, TNM stage, survival time, and survival status. Patients with incomplete information were excluded from our analysis. Samples with OS ≤ 30 days were excluded for non-neoplastic death (Table 1). Corresponding ferroptosis-related genes were downloaded from the FerrDb database [16].FerrDb is a comprehensive, manually curated, and up-to-date database for studying ferroptosis markers and regulators in health and disease. In this study, we identified 247 genes related to triggering effects (Table S1). The relationship between the ferroptosis-related lncRNAs and BCa was assessed using Pearson correlation. The association was considered significant if the correlation coefficient |R2|> 0.3 at *P* < 0.001. Statistical significance of differential expression of ferroptosis-related lncRNAs was set at a fold-change (*FC*) value of > 1.0 and *FDR*-corrected value of *P* < *0.01*.

**Table 1:** The clinical characteristics of patients in the TCGA dataset

Variable	Number of samples
Age at diagnosis	
≤ 65/ > 65	160/237
gender	
Male/Female	294/103
Grade	
Low Grade/High Grade/NA	18/376/3
stage	
I/II/III/IV/NA	2/124/136/133/2
T	
T0/T1/T2/T3/T4/NA	1/3/114/191/57/31
M	
M0/M1/NA	187/10/200
N	
N0/N1/N2/N3/NA	228/45/76/8/40

### Enrichment Analysis of ferroptosis-related DEGs

Functional enrichment analysis of differentially expressed genes (DEGs) was performed using *Metascape* (http://metascape.org) [17] and the Database for Annotation, Visualization and Integrated Discovery (*DAVID*) [18]. In addition, functional analysis of biological processes (BP), molecular functions (MF), and cellular components (CC) regulated by the differentially expressed ferroptosis-related lncRNAs were analyzed based on Kyoto Encyclopedia of Genes and Genomes (KEGG) data [19] using R software and *Metascape database*. The P-value was set at *P* < 0.05 as a critical value.

### Development of the ferroptosis-related lncRNAs prognostic signature

Least absolute shrinkage and selection operator (LASSO, Tibshirani,1996) method is a compression estimation method, which shapes a more refined model by constructing a penalty function that compresses some coefficients and sets some coefficients to zero. LASSO method retains the advantage of subset shrinkage, and it is also a biased estimation for multicollinear data. Thus, it can realize the selection of variables while estimating parameters, and better solves the multicollinearity problem in regression analysis. We employed LASSO-penalized Cox regression analysis and Univariate Cox regression analysis using the "*glmnet*" package in R to develop the ferroptosis-related lncRNAs signature. The risk score was calculated with the below formula [20], and each BCa patient's risk score was evaluated. With the median value as a bound, the RNAs were divided into low-risk (< median value) and high-risk (≥ median value) groups.

(Coefficient lncRNA_1_ × expression of lncRNA_1_) + (Coefficient lncRNA_2_ × expression of lncRNA_2_) + ⋯ + (Coefficient lncRNA_n_ × expression lncRNA_n_).

### The predictive nomogram

We performed Gene set enrichment analyses (GSEA [21] to define the lncRNAs signatures in the KEGG pathways and searched in the TCGA-BLCA database. Statistical significance was set at *P* < 0.05 and false discovery rate (FDR) of q < 0.25. In order to enable clinicians to easily use the prognostic model to evaluate the 1-year, 3-year and 5-year OS of patients with BCa, We combined univariate and multivariate clinical features(gender、grader、age and stage) with significant prognosis and prognostic models, and established nomogram with R software package regplot (https://github.com/cran/regplot).

### Immunity analysis and gene expression

The CIBERSORT [22], ESTIMATE [23], MCPcounter [24], single-sample gene set enrichment analysis (ssGSEA) [25] and TIMER [26] algorithms were compared to assess cellular components or cell immune responses between high-risk and low-risk groups based on the ferroptosis-related lncRNAs signature. Differences in immune response under different algorithms were revealed using a Heatmap. In addition, ssGSEA was used to quantify tumor-infiltrating immune cell subgroups between the two groups and assess their immune function. The potential immune checkpoint was also acquired from previous literature.

### Cell culture

Bladder cancer cell lines T24 and EJ and normal bladder epithelial cells line SV-HUC were obtained from the Shanghai Branch, Chinese Academy of Sciences. The cells were cultured in RPMI-1640 (ThermoFischer Scientific, Waltham, MA, USA) with 10% fetal calf serum (Sigma-Aldrich, St. Louis, MO, USA) and passaged by 0.25% trypsinization with EDTA (Invitrogen, Grand Island, NY). All the cells were cultured at 37° C in 5% CO_2_.Tumor cells in logarithmic phase were selected for experiment.

### Validation of the diff-lncRNAs

The identified diff-LncRNAs were further validated by real-time-quantitative PCR (RT-qPCR) analysis using the following human cell lines (Bladder cancer cells (T24 and EJ) and normal bladder epithelial cells line SV-HUC). The cell lines were purchased from the Shanghai Institute of Cell Science, Chinese Academy of Sciences. The RT-qPCR was conducted according to the procedures. Briefly, TRIzol (Invitrogen; Thermo Fisher Scientific, Inc., Carlsbad, CA, USA) was used to extract total RNA from the cells. The lncRNAs primers and real-time fluorescent quantitative PCR testing kit were obtained from FulenGen Co., Ltd. (Guangzhou, China). 18S served as an endogenous control. The relative quantification of LncRNA levels was determined by the △△C t method. The synthesis of our first cDNA was synthesized using EntiLink™ 1st Strand cDNA Synthesis Kit (ELK Biotechnology, EQ003), and real-time fluorescent quantitative PCR was conducted on the StepOne™ Real-Time PCR instrument (Life Technologies) using EnTurbo™ SYBR Green PCR SuperMix kit (ELK Biotechnology, EQ001), and 3 double holes were set up for each sample. The specific primer sequence list was shown in Table 2.

**Table 2:** The specific primer sequence list

Gene name	sequence
AL031775.1	Forward GGTGTCTGTATATTGTCCTATCCA Reverse CAGTCCATCATCAAGATTGTAAGG
AC018653.3	Forward CTACCTGTCCTGCCTCCTTC Reverse GCCCATGCTTTCCAGATGTATT
AC011468.1	Forward AAGCAGTATTTCGGAAGCACTT Reverse AACTCCTGACTTCTAGGTTGAGA
AL583785.1	Forward CTGGGAAAGGCAAGGATGTG Reverse ACAAACTGGGTGGCTTACAAC
AC021321.1	Forward GCATCTGTCACTGTTCTGTTCTAT Reverse TAGCCTTCCTAAATCTGGTCACT
AP003352.1	Forward TGCCTCAGCCTCTCAAGTAG Reverse CGTGGCTCACACCTGTAATC
`ETV7-AS1`	Forward CAACGGTGCTAGTGGTAGTAGT Reverse GCTTCTCCTTCTCGGTGACA
U47924.1	Forward AATGGGTGATGTGGGAGAAATG Reverse TCCTGGGTCTGTCTTTGTGT
AC010326.3	Forward GCTTCCGAGATCAGACGAGAT Reverse TCAAAGAGAGATGCCACACATTG
LINC02762	Forward CATTCAGGCAGTCAGCACAA Reverse TGAGGCAGGAGAATCACTTGA
18S	Forward CACCAGACTTGCCCTCCAAT Reverse CCTGAGAAACGGCTACCACAT

### Drug sensitivity analysis

We use the pRRophetic algorithm [27] to predict the IC50 value of drugs by constructing a relevant ridge regression model. The model takes the expression profile of GDSC cell line(https://www.cancerrxgene.org/) as the training set and TCGA queue as the validation set. we predicted the IC50 values of axitinib, bortezomib, cisplatin, gefitinib, sorafenib, sunitinib, temsirolimus and vinblastine drugs in each sample of TCGA data set. Spearman correlation test was used to analyze the correlation between the expression of lncRNA and the IC50 values of these drugs and cisplatin.

### Statistical analysis

Data were analyzed using Bioconductor packages in R software (version 4.0.2). Normal and non-normal distributed variables were analyzed by the unpaired student's t-test and the Wilcoxon test, respectively. Benjamini–Hochberg method was used to identify the differentially expressed lncRNAs based on FDR. The ssGSEA-normalized BCa DEGs were compared with a human genome using "GSVA" (R-package). The sensitivity and specificity of the derived prognostic signatures for BCa in comparison to other clinicopathological was assessed by the receiver operating characteristic (ROC) curve and decision curve analysis (DCA) [28]. The relationship between ferroptosis-related lncRNAs and clinicopathological manifestations was evaluated using logistic regression analyses and a heatmap graph. Finally, the survival analysis of BCa patients based on the ferroptosis-related lncRNAs signature was analzyed using the Kaplan–Meier survival analysis. For each analysis, statistical significance was set at *P* < 0.05.

## Results

### Extraction and functional enrichment analysis of differential genes associated with ferroptosis

**Fig. 1:**
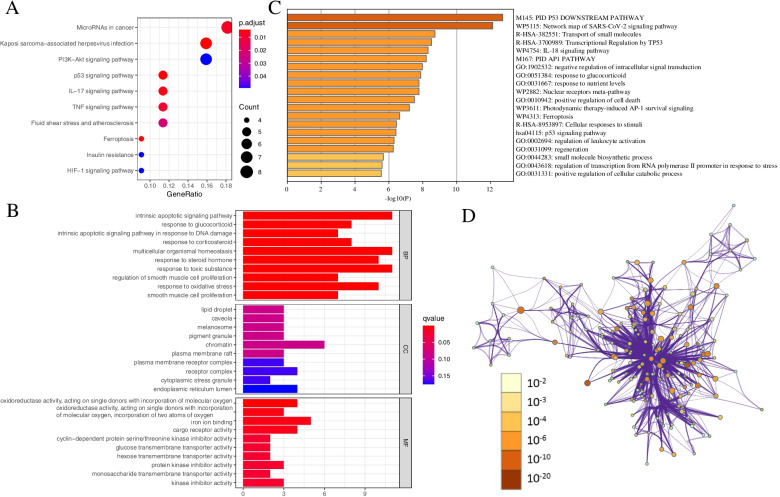
GO and KEGG analyses for ferroptosis-related differentially expressed genes. (**A**) KEGG. (**B**) GO. (**C**-**D**) GO and KEGG analyses for ferroptosis-related differentially expressed genes by metascape

We began with analyzing the BCa transcriptome expression data obtained from *TCGA*. By differential enrichment analysis using DAVID database, 61 differentially expressed genes (DEGs) were found to be associated with ferroptosis. Of these genes, 25 were downregulated, and 36 were upregulated (Table S2). BP functional enrichment analysis revealed that these genes were involved in intrinsic apoptotic signaling pathway, multicellular organismal homeostasis, and response to toxic substances. Among the MF specific terms, we found “iron ion binding”, “cargo recrptor activity”, “oxidoreductase activity acting on single donors with incorporation of molecular oxygen”, and “protein kinase inhibitor activity”. Among the CC terms, we mainly found “lipid droplet”, “caveola”, and “chromatin”. KEGG-based analysis showed that overexpressed genes mainly involved the p53 signaling pathway, ferroptosis, Kaposi sarcoma-associated herpesvirus infection, IL-17 signaling pathway, MicroRNAs in cancer, the TNF signaling pathway, PI3K-Akt signaling pathway, and HIF-1 signaling pathway (Fig. 1A-D).

### Prognostic features and survival analysis based on ferroptosis-associated lncRNAs

In this study, 518 ferroptosis-related lncRNAs were identified(Table S3), and 34 lncRNAs associated with the prognosis of bladder cancer were screened out by univariate Cox analysis. The result showed that except for AL583785.1 and LINC02762 which were high-risk prognostic LncRNAs, others were low-risk LncRNAs( *P* < 0.05, Fig. 3C). Multivariate Cox analysis of these obtained lncRNAs showed that 7 of the 11 differentially expressed lncRNAs (Table S4) were independent prognostic indicators of BCa.

**Fig. 2:**
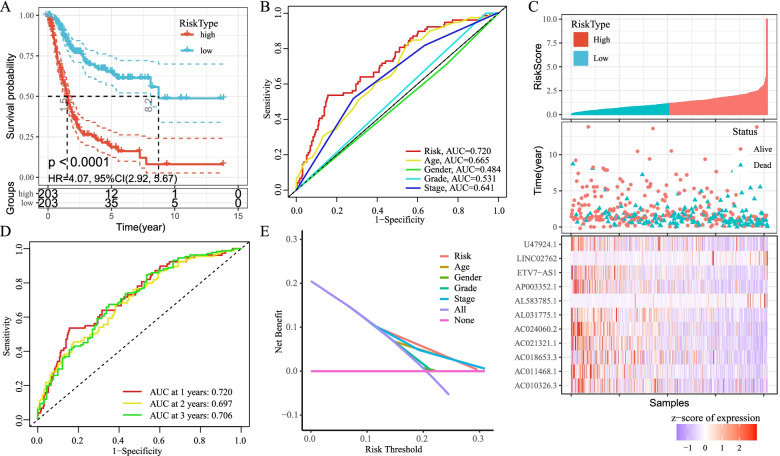
Ferroptosis-related lncRNAs signature based on TCGA. **A** Kaplan–Meier curves result. **B** The AUC values of the risk factors. **C** Risk survival status plot. **D** The AUC of the for the prediction of 1, 3, 5-year survival rate of BCa. **E** The DCA of the risk factors

**Fig. 3:**
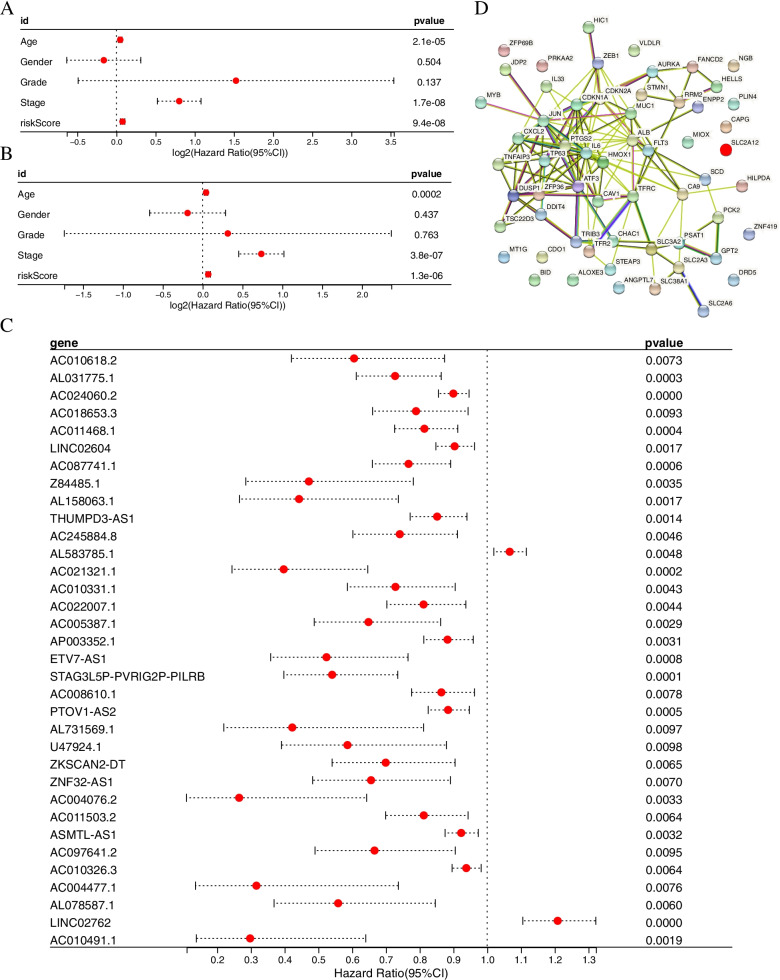
Univariate and multivariate COX analysis for the expression of ferroptosis-related lncRNAs. (**A**) Univariate. (**B**) Multivariate. (**C**) Univariate COX analysis for the expression of ferroptosis-related lncRNAs. (**D**) The relationship between the novel lncRNA and mRNA expression

**Fig. 4:**
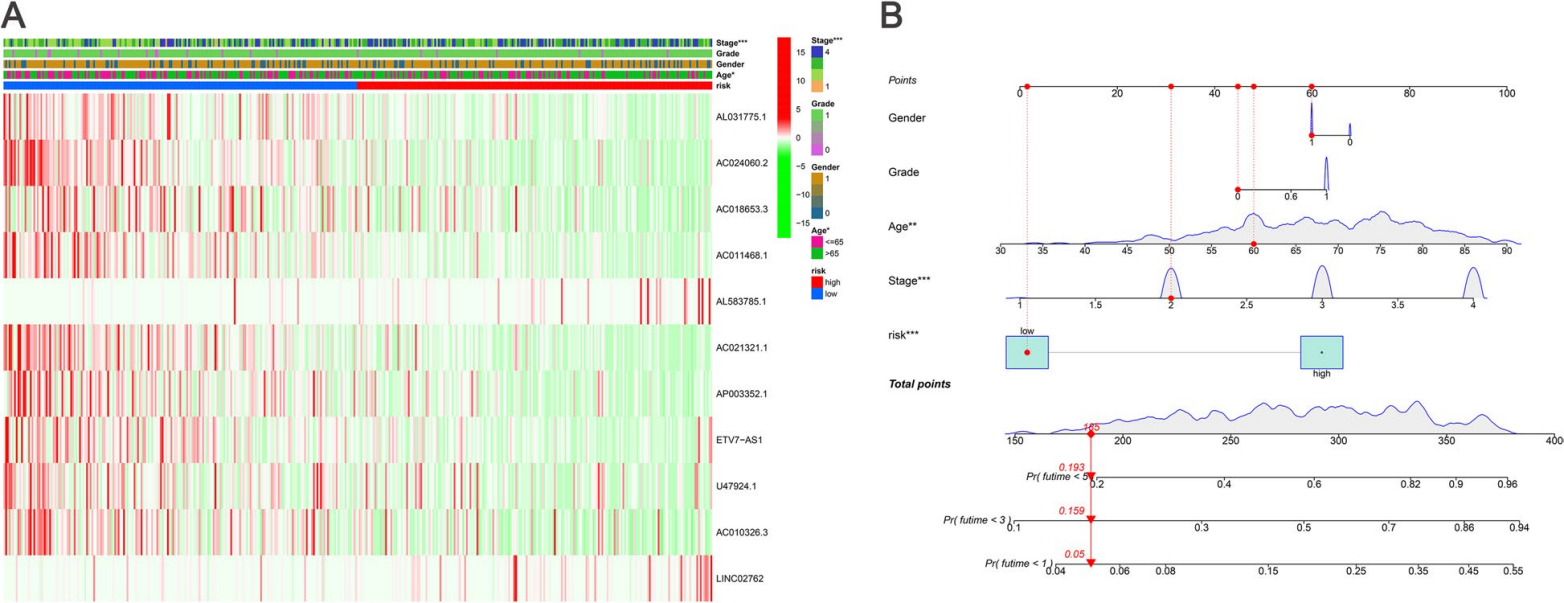
**A** Heatmap for ferroptosis-related lncRNAs prognostic signature and clinicopathological manifestations. **B** A nomogram for both clinic-pathological factors and prognostic ferroptosis-related lncRNAs

We next calculated risk scores and constructed a BCa prognostic model using the lncRNAs. Kaplan–Meier analysis showed that patients with high-risk lncRNAs expression had poorer survival than lncRNAs with low-risk group (*P* < 0.001, Fig. 2A). The risk model (AUC = 0.720) showed a stronger performance and predictive power than traditional clinicopathological features (Fig. 2B). Using the patients' risk survival status maps, we found that patients' risk scores were inversely correlated with the survival of BCa patients. Interestingly, from the heat map, we found that most of the novel lncRNAs identified in this study were negatively correlated with the risk model (Fig. 2C). The AUC predictive values of these new IncRNA models for 1-year, 3-year, and 5-year survival were 0.720, 0.697, and 0.706, respectively (Fig. 2D).The DCA plot showed the optimal predictive performance of our risk model (Fig. 2E). The heat map showed the visualization of the expression of the 11 ferroptosis-related lncRNAs included in the risk model (Fig. 2C). Univariate and multifactorial Cox analyses showed that lncRNAs model (HR: 1.05, 95CI: 1.03–1.07) and tumor stage (HR: 1.66, 95CI: 1.37–2.03) were independent prognostic factors for OS of BCa patients (Fig. 3A-B). Figure 3D shows the interactions of prognosis-related LncRNAs regulating genes. A heatmap of the association between prognostic model and clinicopathological manifestations of lncRNAs associated with ferroptosis was also analyzed (Fig. 4A). A hybrid column line plot combining clinicopathological characteristics and the novel ferroptosis-related lncRNAs prognostic signature (Fig. 4B) was stable and accurate, therefore it could be applied in clinical management of BCa patients.

### Gene set enrichment analysis

**Fig. 5:**
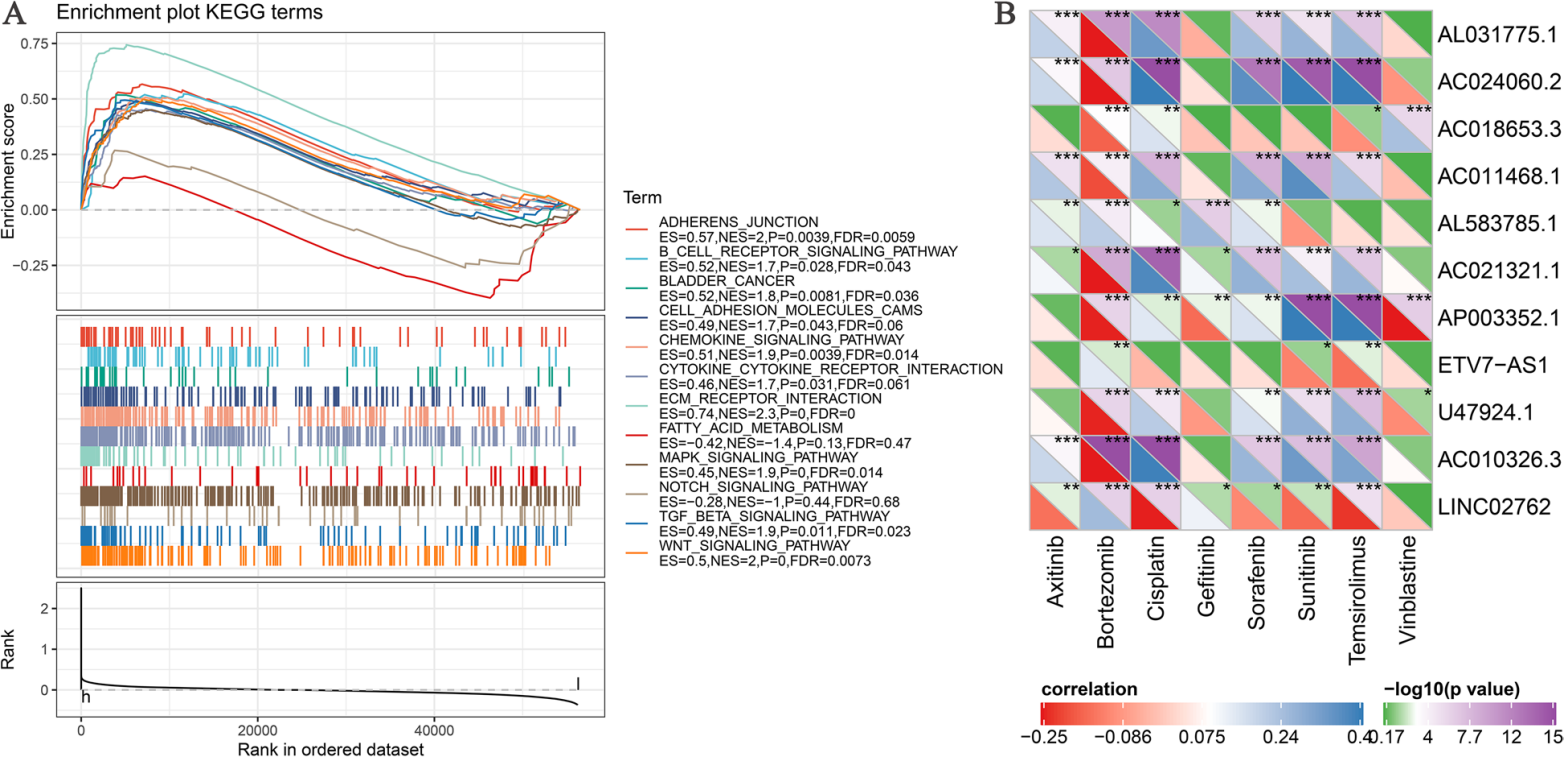
**A** Gene enrichment analysis for ferroptosis-related lncRNAs based on TCGA. **B** Correlation between the expression of 11 lncRNAs and drugs

The gene set enrichment analysis (GSEA) revealed various immune and tumor-related pathways, which are prognostic signature regulators of most novel lncRNAs associated with ferroptosis, such as Adhesion junction, ECM receptor interaction, Chemokine signaling pathway, B cell receptor signaling pathway, TGF-β signaling pathway, MAPK receptor signaling pathway, Notch signaling pathway, and Bladder cancer. (Fig. 5A). Using the R software package pRRophetic, we predicted the IC50 values of axitinib, bortezomib, cisplatin, gefitinib, sorafenib, sunitinib, temsirolimus and vinblastine drugs in each sample of TCGA data set, and further analyzed the Spearman rank correlation coefficient between the expression of these lncRNAs and these drugs. It can be observed that multiple lncRNAs have significant correlation with the IC50 of various drugs, Bortezomib showed significant negative correlation with eight lncRNAs (Fig. 5B).

### Expression of immune-related genes

**Fig. 6:**
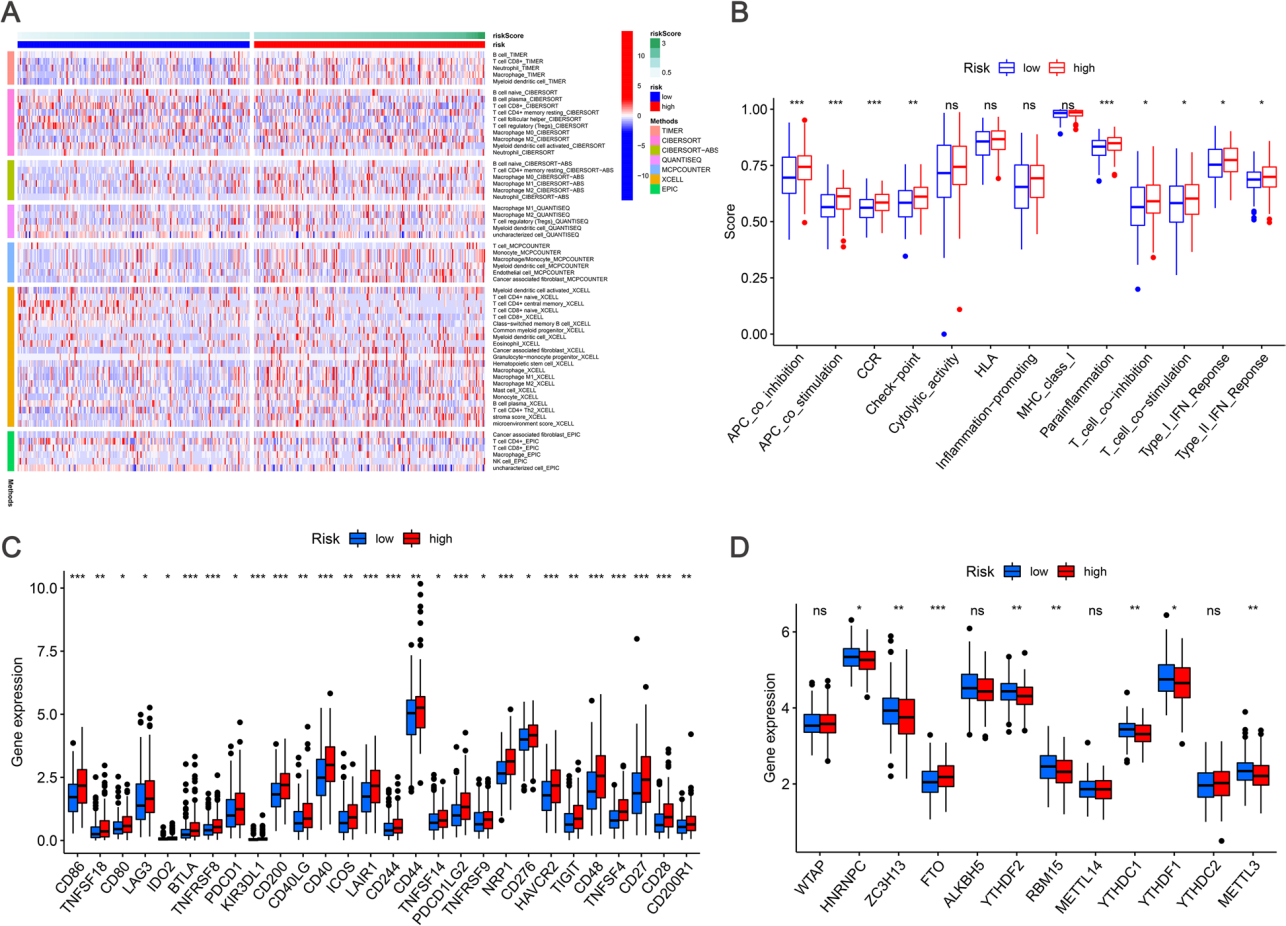
**A** Heatmap for immune responses based on CIBERSORT, ESTIMATE, MCPcounter, ssGSEA, and TIMER algorithms among high- and low-risk group. **B** ssGSEA for the association between immune cell subpopulations and related functions **C**. Expression of immune checkpoints among high and low BCa risk groups. **D** The expression of m6A-related genes between high and low BCa risk group

Next, we generated a heat map of the immune response based on CIBERSORT, ESTIMATE, MCPcounter, single sample gene set enrichment analysis (ssGSEA), and TIMER algorithm (Fig. 6A). From ssGSEA on the TCGA-BLCA data and correlation analysis between immune cell subsets and related functions, it was found that T cell functions included checkpoint (suppression), lysis, HLA, inflammatory regulation, co-stimulation, co-inhibition, and type II INF response. Significant differences between low-risk and high-risk patients were detected (Fig. 6B). As checkpoint inhibitor-based immunotherapy strategies are emerging as one of the most promising cancer treatment tools for some drug-resistant tumors, we further explored the differences in immune checkpoint expression between the two groups, and observed significant differences in PDCD-1 (PD-1), CTLA4, LAG3, and BTLA expression (Fig. 6C). In addition, the comparison of m^6^A-related mRNA expression and the expression of METTL3, RBM15, ZC3H13, YTHDC1, YTHDF1, YTHDF2, HNRNP and FTO in the high-risk and low-risk groups were significant (Fig. 6D).

### Validation of the identified Diff-lncRNAs

**Fig. 7:**
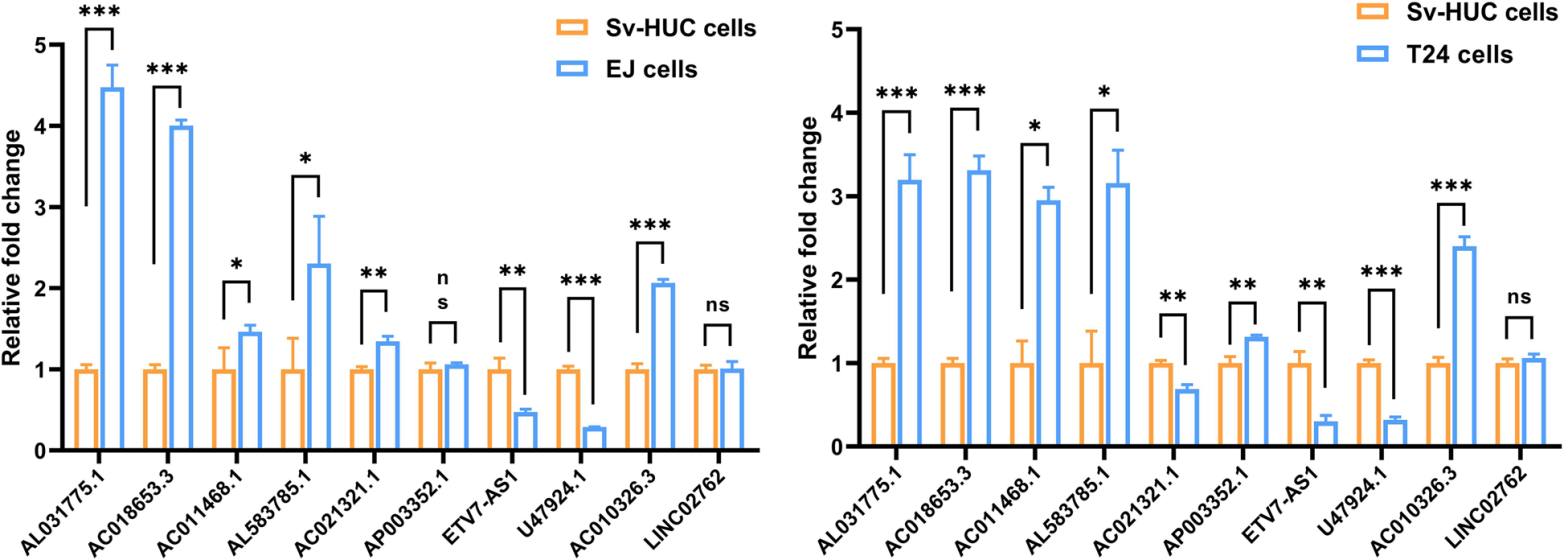
The results of qRT-PCR shows the differential expression of iron death-associated LncRNAs associated with bladder cancer prognosis between bladder cancer cells (T24 and EJ cell lines) and normal bladder epithelial cells line SV-HUC. (* Means *p* < 0.05, ** means *p* < 0.001, *** means *p* < 0.0001)

Figure 7 showed the results of the qRT-PCR. The expression of lncRNAs (AL031775.1, AC018653.3, AC011468.1, AL583785.1, AC021321.1, AP003352.1, `ETV7-AS1`, U47924.1, AC010326.3) were significantly downregulated in the SV-HUC cell line as compared with the T24 cell line, while LINC02762 expression did not show significant differences in the two cells line. Similarly, the expression of lncRNAs (AL031775.1, AC018653.3, AC011468.1, AL583785.1, AC021321.1, `ETV7-AS1`, U47924.1, AC010326.3) was significantly upregulated in EJ cell lines, different from SV-HUC cells. However, the expressions of AP003352.1 and LINC02762 did not show significant differences in the two cell lines. The result indicated that the eight lncRNAs (AL031775.1, AC018653.3, AC011468.1, AL583785.1, AC021321.1, `ETV7-AS1`, U47924.1, AC010326.3) could be used as ferroptosis-related biomarkers to predict the prognosis of BCa patients. Those results provided new targets for the treatment and management of BCa patients. (The specific primer sequences are listed in Table 2).

## Discussion

Ferroptosis or iron-dependent cell death can regulate tumor proliferation, invasion, and progression [29]. Therefore, ferroptosis induction is emerging as a potential anti-cancer therapeutic strategy via triggering tumor cell death, especially for patients with drug-resistant tumors [29]. In this study, we identified 47 DEGs associated with ferroptosis. GO analysis showed that these DEGs were involved in the BP intrinsic apoptotic signaling pathway, multicellular organismal homeostasis, and response to toxic substances. MF terms were mainly related to “regulate iron ion binding”, “oxidoreductase activity”, “acting on single donors with incorporation”, and CC terms were related to chromatin, receptor complex, and endoplasmic reticulum lumen. KEGG signaling pathway prediction analysis further demonstrated that these overexpressed genes were mainly involved in the p53 signaling pathway, Ferroptosis, Kaposi sarcoma-associated herpesvirus infection, IL -17 signaling pathway, MicroRNAs in cancer, TNF signaling pathway, PI3K-Akt signaling pathway, and HIF-1. A recent study showed that p53 could enhance SLC7A11 (solute carrier family 7 member 11), SAT1 (spermidine/spermine N1-acetyltransferase 1), and GLS2 (glutaminase 2) expression by suppressing ferroptosis. On the other hand, p53 inhibits ferroptosis by directly inhibiting DPP4 (dipeptidyl peptidase 4) activity or inducing CDKN1A/p21 (cell cycle protein-dependent kinase inhibitor 1A) expression [30]. Another independent study also confirmed that ferroptosis could be regulated by p53 signaling and tumor-associated mutant p53 (mutp53). The primary manifestation is that the regulation of ferroptosis via p53 contributes to the tumor-suppressive function of p53 and in addition, the accumulation of mutp53 protein in cancer cells increases the sensitivity of cancer cells to ferroptosis [31]. Pretreatment with FG-4592, an inhibitor of prolyl hydroxylase of HIF, reduces renal injury via AKT/GSK-3β-mediated activation of Nrf2 in advanced stages of ferroptosis [32]. In addition, Yadong Sun et al. reported that cancer spheroids proliferate using mammalian targets of rapamycin (mTOR) and utilize the lipid peroxidase GPX4 against ferroptosis [33].

To date, several studies have shown that lncRNAs can act as anti-cancer targets by regulating ferroptosis [34–36]. Ferroptosis-related lncRNAs could predict the prognosis of colon cancer patients [37]. LINC00618 expedites ferroptosis through adding lipid reactive oxygen and iron in leukemia and reduces the level of SLC7A11, which accelerates ferroptosis by inducing apoptosis [38]. A recent independent study suggested that iron-dependent cell death-associated lncRNAs can be a prognostic factor for colorectal cancer and HNSCC patients [39, 40]. Therefore, it is of great significance to developed a ferroptosis-related lncRNA prediction signature for BCa patients. In our present study, we constructed a model characterized by 11 lncRNAs (AL031775.1, AC024060.2, AC018653.3, AC011468.1, AL583785.1, AC021321.1, AP003352.1, `ETV7-AS1`, U47924.1, AC010326.3, and LINC02762) associated with iron-dependent cell death to predict the prognosis of BCa patients. Currently, multiple ferroptosis-related lncRNAs have been reported to be associated with a poor prognosis in a variety of tumors. Mei Chen et al. found that AL031775.1, AP003352.1 could estimate the prognosis and development of bladder cancer patients [41]. AC018653.3 was a gene in fifteen kinds of lncRNAs to predict prognosis in colorectal cancer [42]. Six immune-related lncRNAs, including AC011468.1, showed an underlying significance in the prognosis of bladder cancer patients [43]. For seven remaining ferroptosis-related lncRNAs (AC024060.2, AL583785.1, AC021321.1, `ETV7-AS1`, U47924.1, AC010326.3 and LINC02762), there were no studies reporting their prognostic roles in cancers. Therefore, more studies are needed to explore how these lncRNAs affect the prognosis of BC patients through iron failure.

Subsequently, using GSEA, we revealed potential signaling pathways, for example, Adhesion junction, ECM receptor interaction, Chemokine signaling pathway, B cell receptor signaling pathway, TGF-β signaling pathway, MAPK receptor signaling pathway, Notch signaling pathway, and Bladder cancer, for the 11 ferroptosis-associated lncRNAs. It was reported that ferroptosis promotes neutrophil adhesion to coronary vascular endothelial cells via the TLR4/Trif/I type IFN signaling pathway, thereby coordinating neutrophil recruitment to damaged myocardium and promoting programmed cell death [44]. Several recent studies have confirmed the interaction among ferroptosis and immune checkpoint inhibitors and immune cell infiltration [45]. Similarly, lncRNAs play critical roles in ferroptosis. Previous study found that LINC00618 promotes VCR-induced ferroptosis and apoptosis, while LINC00618 accelerates ferroptosis in an apoptosis-dependent manner. LINC00618 attenuated lymphatic-specific decapping enzyme (LSH) expression and LSH enhanced SLC7A11 promoter region after recruitment to SLC7A11 transcription, further inhibiting ferroptosis [38]. LncRNA OIP5-AS1 promotes PCa progression and ferroptosis resistance through miR-128-3p/SLC7A11 signaling [46]. More interestingly, we also discovered a relationship between ferroptosis-associated lncRNAs and m^6^A methylation genes.

Despite the encouraging data, certain key questions such as the interconnection of ferroptosis with other types of cell death and host immunogenicity remain unclear. This study discovered novel ferroptosis biomarkers for BCa prognosis. These biomarkers could be used to predict and potentially treat BCa. The signature profile should be further investigated using a different cohort as our findings were not validated using clinical samples. Therefore, an in-deep validation with more clinical data from BCa patients is needed before translating our results into clinical practice.

## Conclusion

In summary, we developed a BCa prognostic model base on eleven ferroptosis-related genes (AL031775.1, AC024060.2, AC018653.3, AC011468.1, AL583785.1, AC021321.1, AP003352.1`ETV7-AS1`, U47924.1, AC010326.3 and LINC02762). The novel model lays a foundation for further developing new research strategies to explore the mechanisms of ferroptosis and predicting the prognosis of BCa patients.

## Supplementary Information

**Additional file 1.**

**Additional file 2.**

**Additional file 3.**

**Additional file 4.**

## Data Availability

The datasets generated and/or analyzed during the current study are available in the [https://portal.gdc.cancer.gov/].

## Abbreviations

BCa: Bladder cancer
TCGA: The cancer genome atlas
diff-LncRNAs: Differentially expressed LncRNAs
qRT-PCR: Real-time quantitative PCR
GO: Gene Ontology
KEGG: Kyoto Encyclopedia of Genes and Genomes
CC: Cellular component
MF: Molecular function
BP: Biological process
FC: Fold-change
DEGs: Differentially expressed genes
GSEA: Gene Set Enrichment Analysis
DAVID: The Database for Annotation, Visualization and Integrated Discovery
OS: Overall Survival
ssGSEA: Single-sample gene set enrichment analysis
EDTA: Ethylene Diamine Tetraacetic Acid
DCA: Decision curve analysis
